# Real-World Distributions and Concordance of C-Reactive Protein and Erythrocyte Sedimentation Rate Across Rheumatic Diseases

**DOI:** 10.3390/clinpract16040072

**Published:** 2026-04-13

**Authors:** Claudiu C. Popescu, Luminița Enache, Carmen Ștențel, Corina Mogoșan, Cătălin Codreanu

**Affiliations:** 1 Rheumatology Department, Carol Davila University of Medicine and Pharmacy, 020021 Bucharest, Romania; claudiu.popescu@reumatologiedrstoia.ro (C.C.P.); carmen.stentel@reumatologiedrstoia.ro (C.Ș.); corina.mogosan@reumatologiedrstoia.ro (C.M.); catalin.codreanu@reumatologiedrstoia.ro (C.C.); 2 Clinical Centre for Rheumatic Diseases, 020983 Bucharest, Romania

**Keywords:** C-reactive protein, erythrocyte sedimentation rate, rheumatic diseases

## Abstract

**Objective**: The objective of this study was to characterize real-world distributions of C-reactive protein (CRP) and erythrocyte sedimentation rate (ESR) across major rheumatic diagnoses and to quantify concordance/discordance patterns and combined CRP-ESR inflammatory phenotypes. **Methods**: We retrospectively extracted all CRP and ESR tests performed in a tertiary university rheumatology hospital (January 2018–December 2023), including ICD-10-coded diagnoses. Analyses were conducted at the measurement level and patient level (medians across repeated tests). CRP and ESR were expressed as raw values and multiples of ULN and categorized into severity strata. CRP and ESR datasets were merged by patient identifier and calendar date to define same-day pairs; paired analyses used Spearman correlations and ULN-based phenotype classes. Sensitivity analyses tested alternative pairing windows, first-pair-only analyses, phenotype persistence rules, and tertile/quartile discordance definitions. **Results**: Among 16,921 patients with ≥1 CRP and 17,126 with ≥1 ESR, CRP was more disease-discriminative and only negligibly age-related, whereas ESR increased modestly with age and showed marked sex shifts across severity categories. Inflammatory burden was highest in gout and rheumatoid arthritis, intermediate in psoriatic arthritis and ankylosing spondylitis, and lower in connective tissue diseases (systemic lupus erythematosus, mixed connective tissue disease, Sjogren’s disease, systemic sclerosis, and dermato/polymyositis) and osteoarthritis; CRP distributions were more strongly right-tailed than ESR. Merging yielded 44,427 same-day CRP-ESR pairs from 16,824 patients (99.1% match). CRP and ESR were moderately correlated at measurement and patient levels, yet discordance was common: 27.3% of pairs showed isolated elevation of a single marker. **Conclusions**: In routine rheumatology care, CRP and ESR provide complementary information. CRP-ESR dissociation is frequent, persists at the patient level, and follows diagnosis-dependent phenotype patterns.

## 1. Introduction

C-reactive protein (CRP) and erythrocyte sedimentation rate (ESR) remain central to disease activity assessment, classification, monitoring, and prognosis in rheumatology. They are inexpensive and widely available yet reflect distinct biology: CRP is a hepatic acute-phase protein induced primarily by interleukin-6, whereas ESR is an indirect composite influenced by plasma proteins, red blood cell properties, hematocrit, age, and sex. Consequently, CRP and ESR are related but non-interchangeable; simultaneous ordering may be redundant in some infectious settings [1,2,3], motivating de-implementation strategies [4], but across rheumatic diseases, they are better viewed as complementary markers. Discordance is common and clinically informative: both markers associate with active rheumatic disease [5] or specific manifestations [6], and discrepant phenotypes may carry prognostic signals [7]. In rheumatoid arthritis (RA), DAS28-ESR and DAS28-CRP correlate at the group level yet misclassify individuals, with DAS28-ESR systematically higher, particularly in older women and long-standing disease [8,9,10,11]. In connective tissue diseases, such as systemic lupus erythematosus (SLE), both markers may rise in flare or infection [12,13]; CRP increases more strongly with lupus arthritis [14], and a higher ESR/CRP ratio favors flare over infection in febrile patients [15]. In axial spondyloarthritis, both markers have limited sensitivity for magnetic resonance imaging (MRI)-defined inflammation [16]; ESR may relate more broadly to function/impact [17], while CRP may better discriminate early or untreated disease [18]. Uncertainty is amplified by normal markers in classical inflammatory disease at diagnosis (e.g., polymyalgia rheumatica) [19,20] and by elevated markers in patients without known inflammatory disease [21,22].

Large, real-world, multi-diagnosis studies simultaneously examining CRP and ESR at both measurement and patient levels and explicitly quantifying stable combined phenotypes across repeated testing remain scarce. Demographic effects (age/sex), disease casemix, and repeated longitudinal measurements further shape distributions and concordance patterns in tertiary rheumatology but are insufficiently characterized. Therefore, the study aimed to describe demographic and disease-specific CRP and ESR distributions; compare biomarker behavior across inflammatory and non-inflammatory rheumatic diagnoses; quantify CRP-ESR relationships at measurement and patient levels; and define and quantify combined CRP-ESR inflammatory phenotypes to characterize concordance and discordance in routine care.

## 2. Materials and Methods

### 2.1. Data Sources and Study Population

A retrospective analysis was performed using the electronic laboratory database of a university tertiary rheumatology hospital serving a nationwide referral population. All admissions and day-care visits between January 2018 and December 2023 were retrieved, including age, sex, ICD-10 diagnoses, and CRP/ESR test dates and values. All patients with ≥1 CRP or ESR during the study period were eligible. Standardized clinical disease activity indices, medications (including glucocorticoids), acute infection status, detailed non-rheumatological comorbidities, and setting (inpatient and outpatient) were not available for automatic extraction and therefore could not be analyzed or included as adjustment variables. Identifiers were pseudonymized before analysis. Patients routinely provided written consent for sampling and scientific use of their data; the ethics committee approved the protocol and waived additional consent due to the retrospective, pseudonymized design.

### 2.2. Laboratory Measurements

All assays were performed in the certified hospital laboratory using a single analytical platform and standardized commercial kits under the supervision of the same laboratory physician. CRP and ESR are part of a routine baseline laboratory package performed for all patients presenting to the rheumatology clinic/day-care unit, regardless of diagnosis, including non-inflammatory conditions such as OA. CRP was reported in mg/L with a fixed ULN of 5 mg/L. ESR was reported in mm/h with age- and sex-adjusted ULN provided by the laboratory. Values were additionally expressed as multiples of ULN (“times ULN”); abnormality was defined as >ULN. Severity categories were prespecified based on multiples of ULN, respectively: CRP: 0 (normal), 1 (<2 × ULN), 2 (2-<3 × ULN), 3 (3-<5 × ULN), 4 (5-<10 × ULN), 5 (10-<20 × ULN), 6 (≥20 × ULN); and ESR: 0 (normal), 1 (<2 × ULN), 2 (2-<3 × ULN), 3 (3-<5 × ULN), 4 (≥5 × ULN). Severity strata were defined a priori using multiples of the ULN to place CRP and ESR on a comparable scale despite different units and reference ranges (fixed CRP ULN versus age-/sex-adjusted ESR ULN). ULN-based strata also mirror routine clinical interpretation (normal versus abnormal) and provide a robust ordinal representation for highly right-skewed biomarkers.

### 2.3. Diagnostic Classification

Diagnoses were derived from ICD-10 primary and secondary codes recorded at testing. Because overlap is clinically plausible and primary coding may be influenced by reimbursement, diagnoses were treated as non-mutually exclusive. For each measurement, binary indicators were created for gout, RA, ankylosing spondylitis (AS), psoriatic arthritis (PsA), SLE, mixed connective tissue disease/Sjögren’s disease (MCTD/SjD), systemic sclerosis (SS), dermatomyositis/polymyositis (DM/PM), and osteoarthritis (OA). Overlap was quantified as the row-wise sum of indicators. For analyses requiring mutually exclusive diagnostic strata (e.g., diagnosis-stratified plots and correlations), a single analytic diagnosis per record was assigned deterministically according to a fixed indicator order (gout, RA, AS, PsA, SLE, MCTD/SjD, SS, DM/PM, and OA). This rule served only as a tie-breaker to prevent double-counting across strata and does not imply clinical hierarchy or disease prevalence.

### 2.4. Statistical Management and Analysis

CRP and ESR were available as repeated measurements per patient. Analyses were conducted at the measurement level (each test as an observation) and the patient level (summarizing repeated values per patient using medians). CRP and ESR datasets were merged by patient identifier and calendar date; timestamps containing hours, minutes, seconds, and AM-PM were truncated to month/day/year, and only same-day CRP-ESR pairs were retained. As per institutional workflow, acute-phase reactants are obtained at most once per patient per day (rarely, when multiple same-day blood draws occur, these typically relate to other laboratory panels rather than repeat CRP/ESR measurements); thus, pairing by patient identifier and calendar date was one-to-one, and duplicate-resolution rules (e.g., averaging or selecting earliest/latest) were not required. Merge quality was summarized by the number of paired measurements, the number of unique patients with ≥1 pair, and the matching rate. Associations were quantified using Spearman’s rank correlation (ρ) for raw values and for ordinal severity categories. All paired analyses were stratified by sex and diagnosis; diagnosis-stratified correlations were computed when ≥30 paired measurements were available. Continuous variables are reported as mean ± standard deviation (SD) or median (Q1 and Q3) as appropriate; categorical variables as *n* (%). Group comparisons used Mann–Whitney U (two groups) or Kruskal–Wallis H (≥3 groups); post hoc pairwise comparisons used pairwise Mann–Whitney U with Holm correction. Proportions were compared by χ^2^ tests. There were no missing data (patient ID, sex, age, measurement dates, CRP and ESR values, their ULN, and diagnosis variables were complete). Tests were two-sided with *p* < 0.05. Given a large sample size, interpretation emphasized effect sizes and distributional patterns. Analyses/figures used Python 3.13 (pandas, SciPy, matplotlib).

### 2.5. Sensitivity Analyses Methods

To address minor timing discrepancies, CRP and ESR were additionally paired using a ±1-day window (nearest-neighbor ESR per CRP within ±1 day), repeating correlations and concordance analyses. To reduce the influence of repeated testing and unequal within-person pairs, paired analyses were repeated using only the first same-day CRP-ESR pair per patient. Patient-level phenotypes were also redefined using “ever abnormal” versus “majority abnormal” (>50% pairs abnormal) to assess phenotype persistence. For comparability with prior laboratory database studies, discordance was additionally evaluated using age/sex-stratified tertiles defining “extreme discordance” (highest vs. lowest tertile), following the approach of Costenbader et al. [23], and quartiles derived from each patient’s first pair defining a 2–3 quartile separation, following Feldman et al. [24], applying quartile thresholds to first-pair (patient-level) and all pairs (measurement-level) datasets. Because many patients contributed multiple paired measurements, clustered robustness analyses were performed to account for within-patient correlation. First, measurement-level estimates were recalculated using patient-weighted analyses, assigning each patient equal total weight distributed across their paired observations. Second, we estimated uncertainty using a cluster bootstrap by patient (resampling patients with replacement and retaining all their paired measurements) to obtain 95% confidence intervals (CI) for correlations and phenotype proportions.

## 3. Results

### 3.1. CRP

#### 3.1.1. Unique Patients with CRP Measurements

Among 16,921 patients with ≥1 CRP, mean age was 59.1 ± 14.4 years (Table 1); CRP tests/patient: median 1 (1–3), maximum 44. Men comprised 26.8% and were younger than women (54.8 ± 14.8 vs. 60.0 ± 13.7; MWU = 1.6 × 108, *p* < 10^−10^). OA was most frequent (65.4%), followed by RA (18.2%), AS (6.6%), and MCTD/SjD (4.2%); other diagnoses were less common (gout 2.4%; PsA 1.5%; SLE 1.0%; SS 0.5%; DM/PM 0.1%). Sex distribution differed by diagnosis (χ^2^ = 1487, *p* < 10^−10^), with male predominance in gout (67.0%) and AS (67.2%) and female predominance in RA (82.5%), PsA (62.2%), SLE (89.9%), MCTD/SjD (80.8%), SS (83.8%), DM/PM (88.0%), and OA (75.5%). Age differed across diagnoses (KWH = 780.4, *p* < 10^−10^): AS was younger than all other groups (all *p* < 10^−10^); SLE was younger than RA/OA/gout/DM/PM (all *p* < 10^−10^); RA/gout/OA were older than PsA and MCTD/SjD (all *p* < 10^−10^); and DM/PM was among the oldest, differing from AS/SLE/PsA/MCTD/SjD (all *p* < 0.01). Differences between RA, gout, and OA were smaller but remained significant after correction.

#### 3.1.2. All CRP Measurements

Across 44,850 CRP measurements, abnormality was more frequent in men than women (44.9% vs. 38.1%; χ^2^ = 181, *p* < 10^−10^), and severity categories differed by sex (χ^2^ = 163, *p* < 10^−10^): normal CRP levels (<1 × ULN) were observed in 61.9% of women and 55.1% of men; mild elevations were comparable between women and men, respectively 16.5% vs. 16.1% for 1-<2 × ULN elevations and 7.0% vs. 7.2% for 2-<3 × ULN elevations; all moderate to severe CRP elevations were consistently more prevalent in men than women: 3-<5 × ULN (7.6% vs. 6.1%), 5-<10 × ULN (7.6% vs. 5.1%), 10-<20 × ULN (4.4% vs. 2.4%), and ≥20 × ULN (2.1% vs. 1.0%).

**Figure 1:**
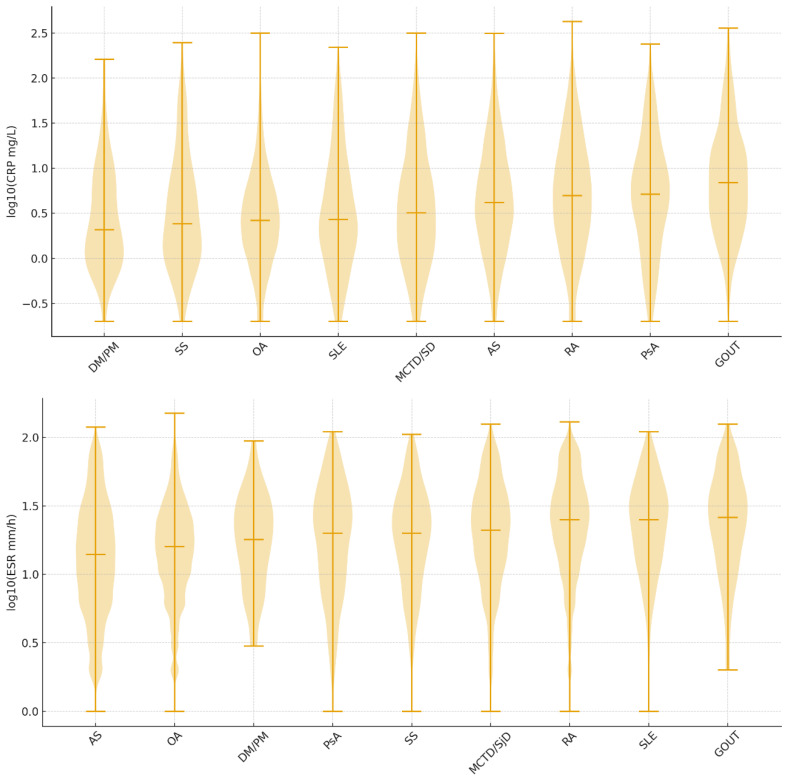
Distribution of CRP values (upper panel; 44,850 measurements) and ESR values (lower panel; 44,627 measurements), displayed as decimal logarithms and medians, among diagnoses. Abbreviations: AS—ankylosing spondylitis; CRP—C-reactive protein; DM/PM—dermatomyositis polymyositis; ESR—erythrocyte sedimentation rate; MCTD—mixed connective tissue disease; OA—osteoarthritis; PsA—psoriatic arthritis; RA—rheumatoid arthritis; SjD—Sjogren’s disease; SLE—systemic lupus erythematosus; SS—systemic sclerosis.

CRP correlated only weakly with age (ρ = 0.053, *p* < 10^−10^). Disease-specific distributions differed markedly (Table 2, Figure 1): gout showed the highest burden (median 6.9 [5.4, 14.2] mg/L; 95th 81.2), RA and PsA were moderate (medians 5.0 and 5.1), AS was slightly lower (4.2), while SLE/MCTD/SjD/SS were predominantly low (medians 2.7–3.2; >60% normal); DM/PM and OA were the lowest. Elevated CRP was most frequent in gout (57.9%) and RA (49.8%) and occurred in 29–39% of AS/OA/CTD measurements; ≥10 × ULN was concentrated in gout (6.1%) and PsA/RA (4–5%). Plots showed strongly right-tailed distributions in gout/RA, intermediate dispersion in PsA/AS, and compact low profiles in CTD/DM/PM/OA.

### 3.2. ESR

#### 3.2.1. Unique Patients with ESR Measurements

Among 17,126 patients with ≥1 ESR, mean age was 59.2 ± 14.4 years; ESR tests/patient: median 1 (1–2), maximum 44. Men comprised 26.8% and were younger than women (56.5 ± 15.2 vs. 60.1 ± 14.0; MWU = 2.47 × 107, *p* < 10^−10^). Diagnosis distribution was similar (OA 66.4%, RA 17.9%, AS 6.5%, MCTD/SjD 4.2%, gout 2.4%, PsA 1.0%, SLE 1.0%, SS 0.5%, and DM/PM 0.1%). Sex differed by diagnosis (χ^2^ = 1497, *p* < 10^−10^) in the same direction as CRP. Age differed across diagnoses (KWH = 781.8, *p* < 10^−10^) with the same pattern as CRP: AS younger than all; SLE younger than RA/OA/gout/DM/PM; RA/gout/OA older than PsA/MCTD/SjD; DM/PM among the oldest.

#### 3.2.2. All ESR Measurements

Across 44,627 ESR measurements, abnormality rates were similar in men and women (42.0% vs. 42.8%; χ^2^ = 2.59, and *p* = 0.11), but severity categories differed strongly by sex (χ^2^ = 1019, *p* < 10^−10^): ESR levels relative to the upper limit of normal (ULN) demonstrated predominantly normal values across both cohorts (<1 × ULN: women 57.2%, men 58.0%); mild elevations (1-<2 × ULN) were more prevalent in women (27.1% vs. 20.2%), whereas moderate elevations (2-<3 × ULN) were comparable (9.7% vs. 9.4%); conversely, higher ESR elevations were disproportionately observed in men (3-<5 × ULN: 9.4% vs. 5.8%; 5-<10 × ULN: 2.9% vs. 0.2%).

ESR showed weak positive age correlations (ESR ρ = 0.236; times-ULN ρ = 0.173; both *p* < 10^−4^). Disease-specific ESR patterns were similar but with narrower high-end ranges than CRP (Table 3, Figure 1): median ESR was highest in gout (26), RA (25), SLE (25), and PsA (20), and it was lower in AS/DM/PM/SS/OA (14–20). The 95th percentile exceeded 70 in gout/RA/PsA/SLE but was lower in AS (66), DM/PM (54), SS (70), and OA (52). Abnormal ESR ranged from 32% (OA) to 61% (gout), with intermediate values in RA/SLE (55%), PsA (47%), and MCTD/SjD (48%). Severe elevations were uncommon (≥5 × ULN: 0.4–3.7%; ≥10 × ULN exceptionally rare). Plots showed broad, moderately right-skewed distributions in gout/RA/SLE/PsA and tighter profiles in DM/PM/SS/OA.

### 3.3. CRP and ESR Pairs

CRP and ESR were merged at the patient-day level, yielding 44,427 same-day pairs from 16,824 patients (99.1% match). Patients contributed a median of 1 pair (1–2; maximum 83); 60.3% had exactly one pair, 39.7% ≥2, and 13.9% ≥5.

#### 3.3.1. Measurement Level

Among pairs, 45.4% were CRP-/ESR- and 27.3% were CRP+/ESR+, while 15.0% were ESR-only and 12.3% were CRP-only. CRP and ESR correlated moderately (ρ = 0.58, *p* < 10^−4^), including across severity categories (ρ = 0.55, *p* < 10^−4^). Correlations were higher in men than women (ρ = 0.67 vs. 0.58; both *p* < 10^−4^) and positive across diagnoses, strongest in DM/PM, AS, gout, and RA (ρ 0.63–0.70) and lower in SLE/SS/OA (ρ 0.44–0.48) (Table 4).

#### 3.3.2. Patient Level

**Figure 2:**
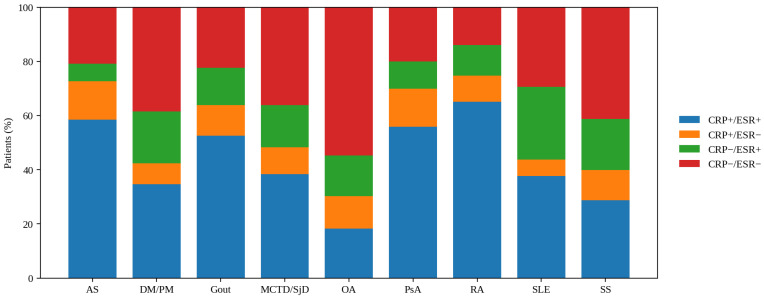
CRP/ESR phenotypes by diagnosis. Stacked bars show the distribution of patient-level CRP/ESR phenotypes among unique patients with ≥1 same-day CRP-ESR pair in each diagnosis (percent within diagnosis). Phenotypes were defined using ULN-based abnormality thresholds (CRP > 5 mg/L; ESR > age- and sex-adjusted ULN): CRP+/ESR+, CRP+/ESR−, CRP−/ESR+, CRP−/ESR−. AS (*n* = 1106): 58.5%, 14.1%, 6.6%, 20.8%; DM/PM (*n* = 26): 34.6%, 7.7%, 19.2%, 38.5%; Gout (*n* = 406): 52.5%, 11.3%, 13.8%, 22.4%; MCTD/SjD (*n* = 714): 38.4%, 9.9%, 15.5%, 36.1%; OA (*n* = 10,935): 18.2%, 12.1%, 15.0%, 54.6%; PsA (*n* = 249): 55.8%, 14.1%, 10.0%, 20.1%; RA (*n* = 3051): 65.1%, 9.6%, 11.3%, 14.0%; SLE (*n* = 167): 37.7%, 6.0%, 26.9%, 29.3%; SS (*n* = 80): 28.7%, 11.2%, 18.8%, and 41.2%. Abbreviations: AS—ankylosing spondylitis; CRP—C-reactive protein; DM/PM—dermatomyositis polymyositis; ESR—erythrocyte sedimentation rate; MCTD—mixed connective tissue disease; OA—osteoarthritis; PsA—psoriatic arthritis; RA—rheumatoid arthritis; SjD—Sjogren’s disease; SLE—systemic lupus erythematosus; SS—systemic sclerosis.

Disease-stratified phenotypes varied (Figure 2): dual-positive predominated in RA/AS/PsA/gout (52.5–65.1%), whereas OA was mostly dual-negative (54.6%) with fewer dual-positive (18.2%); SLE/SS showed relatively higher ESR-only patterns (up to 26.9% in SLE). Median CRP and ESR per patient remained moderately correlated (ρ = 0.54, *p* < 10^−4^), as did median severity categories (ρ = 0.50, *p* < 10^−4^). Patient-level correlations were higher in men than women (ρ = 0.64 vs. 0.53; both *p* < 10^−4^), with diagnosis-specific patterns consistent with measurement-level results.

### 3.4. Diagnosis Overlap

Overlaps were uncommon at the measurement level: 2.62% of CRP and 2.39% of ESR measurements had >1 diagnosis indicator, corresponding to 2.42% and 2.35% of patients with at least one overlapping-coded measurement. Across repeated testing, diagnostic heterogeneity over time was more frequent (CRP 7.19%; ESR 7.45%). Most overlap combinations were rare as patient-level prevalence: RA + MCTD/SjD 0.83%, SLE + MCTD/SjD 0.49%, MCTD/SjD + SS 0.28%, gout + RA 0.20%, RA + SS 0.12%.

### 3.5. Sensitivity Analyses Results

A ±1-day pairing window did not change the paired dataset (44,427 pairs) or associations (ρ = 0.580; severity ρ = 0.546; both *p* < 10^−4^) and preserved concordance (both normal 45.4%, both abnormal 27.3%) and discordance (27.3%; ESR-only 15.0%, CRP-only 12.3%). Restricting to the first pair per patient (*n* = 16,819) versus all complete pairs (*n* = 44,390) yielded similar correlations (ρ = 0.574 vs. 0.580; severity ρ = 0.543 vs. 0.547) and similar concordance patterns. Phenotype persistence definitions shifted absolute proportions but preserved diagnosis-level patterns (ever abnormal: CRP−/ESR− 42.5%, CRP+/ESR− 11.6%, CRP−/ESR+ 13.9%, CRP+/ESR+ 31.9%; majority abnormal: CRP+/ESR+ 19.3%, CRP−/ESR− 53.7%, CRP-only 11.8%, ESR-only 15.2%). Tertile-based extreme discordance occurred in 6.4% of evaluable pairs (3.1% CRP-high/ESR-low; 3.3% ESR-high/CRP-low). Quartile thresholds from first-pair data were CRP Q1 = 1.36, median = 3.14, Q3 = 8.11 mg/L and ESR Q1 = 10, median = 18, Q3 = 30 mm/h; discordance (2–3 quartile separation) occurred in 17.6% of patients (9.5% high CRP/low ESR; 8.1% high ESR/low CRP) and was similar when applied to all pairs (18.4%). An additional repeated paired analysis after excluding patients with any overlap-coded diagnosis indicator was performed. Excluding overlap patients (*n* = 413) did not materially change paired CRP-ESR correlations or concordance patterns (Supplementary Tables S1 and S2).

Clustered robustness analyses supported the measurement-level findings (Supplementary Table S3). Patient-weighted estimates yielded a slightly attenuated CRP-ESR correlation (weighted r = 0.549 versus unweighted 0.580) and shifted phenotype proportions toward more dual-normal pairs (50.9% vs. 45.4%), consistent with frequent testers contributing more abnormal pairs. Cluster bootstrap by patient produced narrow 95% CIs (r = 0.580, 95% CI 0.568–0.594; category r = 0.547, 95% CI 0.532–0.561), and phenotype proportions were similarly stable, indicating that within-patient clustering does not materially alter the main conclusions.

## 4. Discussion

### 4.1. Comparison with Literature

Our real-world same-day paired analysis confirms and extends prior work showing that CRP and ESR are moderately aligned but frequently non-interchangeable in routine care. Using a quartile-based definition in unselected adults from a general hospital laboratory cohort, Feldman et al. [24] reported that paired CRP/ESR results are concordant in approximately 88% of adults and discordant in 12% (6% high CRP/low ESR; 6% high ESR/low CRP), with a moderate Pearson correlation (r = 0.56). Using an analogous approach in our tertiary rheumatology cohort, we observed a higher discordance frequency, while the overall CRP-ESR correlation was similar. Importantly, the directionality of discordance mirrored Feldman’s clinical observations: ESR-high/CRP-low discordance was strongly female-predominant and relatively enriched for connective tissue disease diagnoses, supporting the concept that ESR-only inflammatory patterns cluster in connective tissue diseases, whereas CRP-high/ESR-low discordance appears more typical of non-connective tissue disease inflammatory profiles. In our tertiary rheumatology casemix, concordance (both normal or both abnormal) was 72.7%, and discordant patterns (only one marker abnormal) accounted for 27% of same-day pairs. This higher discordance frequency is directionally consistent with the literature but likely reflects differences in population (rheumatology-enriched versus general hospital) and, critically, discordance definitions. For example, Costenbader et al. [23], in their case–control study of 2069 same-day outpatient pairs, defined discordance more stringently (e.g., “elevated ESR/low CRP” or “elevated CRP/low ESR”, based on opposite tertiles), finding discordance in 4% of patients (2.6% ESR-high/CRP-low; 1.5% CRP-high/ESR-low). By design, our definition based on the ULN captures milder dissociation (e.g., ESR just above the threshold with normal CRP), which is expected to increase the observed discordance rate. Importantly, Costenbader et al. [23] identified infection, renal insufficiency, and low serum albumin as key correlates of discordance, especially for high ESR with low CRP, highlighting that ESR interpretation may be particularly vulnerable to non-inflammatory systemic factors. Our disease-stratified phenotype approach complements this by showing that ESR-only and CRP-only patterns are not randomly distributed across rheumatic diagnoses, supporting the concept that both biological context and systemic modifiers shape CRP-ESR dissociation. More recent work in non-infectious inflammatory diseases emphasizes that CRP-ESR discrepancy is not exceptional but a frequent and definition-dependent phenomenon [25]. This report notes that the most common discordant pattern is elevated ESR with normal CRP and that discordance has been linked to female sex and connective tissue disease contexts, reinforcing that ESR and CRP are complementary rather than interchangeable markers. Our findings closely align with this framework: even with routine ULN-based thresholds, we observed substantial discordance in same-day pairs, and disease-stratified phenotyping showed systematic enrichment of ESR-only profiles in connective tissue diseases (notably SLE/SS), while inflammatory arthritides more often exhibited dual-positive patterns. Therefore, reliance on a single biomarker would miss a non-trivial fraction of inflammatory profiles. Finally, our findings help contextualize primary-care diagnostic literature, questioning the incremental yield of ordering multiple inflammatory markers for “rule-out” purposes. In a large primary-care database analysis, Watson et al. [26] concluded that adding ESR to CRP provided only marginal improvement for ruling out serious disease. Our results do not contradict this: they suggest that the value of dual testing is context-dependent. In rheumatology, the second marker can meaningfully change the inflammatory phenotype (CRP-only versus ESR-only versus dual-positive/dual-negative), and discordance is common enough to raise clinical interpretation issues, particularly when interpreted by diagnosis.

### 4.2. Practical Contributions

This study was undertaken to address a practical gap: despite routine use of CRP and ESR, large real-world datasets rarely quantify how often the two markers agree or diverge across rheumatic diagnoses and whether discordance patterns are stable at the patient level. The study is intended as a descriptive benchmark and hypothesis-generating framework rather than a mechanistic or prognostic analysis. By pairing CRP and ESR at scale on the same patient-day and analyzing results at both measurement and patient levels, the study provides disease-stratified reference distributions and a reproducible CRP-ESR phenotype framework. This framework shows that ESR-only and CRP-only inflammation are not random noise but diagnosis-patterned signatures that persist across sensitivity analyses, supporting complementary interpretation and informing future work linking phenotypes to outcomes.

### 4.3. Limitations

This study has several limitations inherent to real-world laboratory databases. The cohort is derived from a single tertiary rheumatology center and reflects referral and casemix patterns specific to specialized care. Therefore, absolute distributions and phenotype proportions may not generalize to primary care or population-based settings. Diagnoses were assigned from ICD-10 codes recorded in routine practice, which may be influenced by administrative or reimbursement considerations and may allow for misclassification and residual overlap between diagnoses. Analyses of non-unique measurements treat each test as an observation and therefore reflect both biological variability and differences in testing frequency and clinical severity over time. While this was complemented with patient-level medians to reduce within-person clustering, the measurement-level results may over-represent frequently monitored individuals and may overstate nominal precision (*p*-values); therefore, inference is based primarily on effect sizes, patient-level analyses, and robustness checks. The CRP-ESR merge was restricted to same-day pairs using calendar dates, which improves specificity but may under-capture clinically related tests performed on adjacent days; this may particularly affect less frequently monitored diseases and partially explains smaller paired sample sizes in rarer conditions. Because SS and DM/PM counts were small, effect estimates are less stable despite statistical significance. The study did not incorporate key determinants of ESR and CRP behavior, such as hemoglobin, albumin, renal function, infection status, medications (e.g., glucocorticoids), and clinical setting, which may partly explain discordant CRP-ESR phenotypes, thus limiting causal inference about mechanisms underlying discordance. Because of this, the findings should be interpreted as descriptive and hypothesis-generating, since they quantify the frequency and diagnosis-patterning of discordance rather than proving prognostic or therapeutic relevance. The study also lacked contemporaneous clinical disease activity measures (e.g., DAS28, ASDAS/BASDAI, SLEDAI), preventing direct linkage of CRP-ESR phenotypes to clinical activity states at the time of testing. The study design is observational and descriptive; statistical significance is expected in large datasets and does not necessarily imply clinically meaningful differences. The prominence of OA reflects routine universal testing rather than inflammatory case enrichment; therefore, pooled distributions should be interpreted in the context of a real-world referral mix, while diagnosis-stratified analyses provide the primary disease-specific interpretation.

### 4.4. Further Research

Future research should validate these disease-specific distributions and CRP-ESR phenotypes in multicenter cohorts and in population-based datasets. Methodologically, pairing strategies that allow short windows (±24–72 h) or nearest-neighbor matching could be compared against strict same-day pairing to assess robustness and to improve capture in rare diseases. Mechanistic analyses should incorporate hematologic indices, albumin, renal function, and markers of infection, enabling multivariable modeling of CRP-ESR discordance and ESR-only versus CRP-only phenotypes in order to identify independent predictors of CRP-ESR discordance and phenotype persistence. Future studies should also link laboratory data to structured disease activity measures and treatment records to test whether CRP-ESR phenotypes improve discrimination of clinical activity or predict outcomes. Longitudinal studies linking biomarker phenotypes to clinical outcomes would clarify prognostic relevance and inform whether joint CRP-ESR interpretation adds value beyond either marker alone.

## 5. Conclusions

CRP and ESR showed distinct real-world behavior across rheumatic diagnoses and demographic strata. CRP was more disease-discriminative and only negligibly related to age, whereas ESR was more age-dependent and showed marked sex-related shifts in severity categories. Inflammatory burden was highest in gout and RA, intermediate in PsA and AS, and generally lower in connective tissue diseases and OA. CRP distributions were more strongly right-tailed than ESR. In same-day pairs, CRP and ESR were moderately correlated at both measurement and patient levels, yet discordance was common: about one quarter of pairs had isolated elevation of only one marker. Phenotype patterns were disease-specific: dual-positive profiles predominated in inflammatory arthritides, OA was largely dual-negative, and connective tissue diseases (especially SLE/SS) were relatively enriched for ESR-only phenotypes. Sensitivity analyses yielded similar results, supporting that CRP-ESR dissociation is robust and frequent in routine rheumatology care. Unlike prior laboratory database studies focused on extreme discordance, we demonstrate that milder CRP-ESR dissociation is common even around routine ULN thresholds, is stable at the patient level, and follows distinct diagnosis-dependent patterns, providing a practical disease-associated CRP-ESR phenotype framework rather than interchangeable use of the two markers. Mechanistic attribution and prognostic value require outcome-linked validation.

## Figures and Tables

**Table 1:** Diagnosis frequency, age, and sex distribution among unique patients with at least one CRP (*n* = 16,921) or ESR measurement (*n* = 17,126).

CRP Diagnoses	% of *n* = 16,921	Age (years)	Men (%)	Women (%)
OA	65.4%	59.4 ± 14.5	24.5%	75.5%
RA	18.2%	62.4 ± 12.9	17.5%	82.5%
AS	6.6%	49.4 ± 12.8	67.2%	32.8%
MCTD/SjD	4.2%	55.9 ± 17.0	19.2%	80.8%
Gout	2.4%	62.6 ± 11.0	67.0%	33.0%
PsA	1.5%	58.1 ± 12.4	37.8%	62.2%
SLE	1.0%	51.3 ± 13.7	10.1%	89.9%
SS	0.5%	58.0 ± 12.9	16.2%	83.8%
DM/PM	0.1%	64.2 ± 13.4	12.0%	88.0%
ESR Diagnoses	% of *n* = 17,126	Age (years)	Men (%)	Women (%)
OA	66.4%	59.4 ± 14.4	24.5%	75.5%
RA	17.9%	62.4 ± 12.9	17.5%	82.5%
AS	6.5%	49.5 ± 12.8	67.3%	32.7%
MCTD/SjD	4.2%	55.9 ± 17.0	19.0%	81.0%
Gout	2.4%	62.8 ± 11.0	67.1%	32.9%
PsA	1.0%	59.3 ± 11.6	39.1%	60.9%
SLE	1.0%	51.2 ± 13.7	10.1%	89.9%
SS	0.5%	58.0 ± 12.8	16.7%	83.3%
DM/PM	0.1%	64.2 ± 13.4	12.0%	88.0%

Notes: Percentages are calculated within diagnosis categories, and each patient was assigned a single analytic diagnosis to ensure mutually exclusive groups. Age is reported as mean ± SD (years). Abbreviations: AS—ankylosing spondylitis; DM/PM—dermatomyositis/polymyositis; MCTD/SjD—mixed connective tissue disease and Sjögren’s disease; OA—osteoarthritis; PsA—psoriatic arthritis; RA—rheumatoid arthritis; SLE—systemic lupus erythematosus; SS—systemic sclerosis.

**Table 2:** Non-unique CRP distribution among diagnoses (*n* = 44,850 measurements).

	Gout	RA	AS	PsA	SLE	MCTD/SjD	SS	DM/PM	OA
*n*	872	15,794	6430	1197	673	2977	542	200	17,108
mean	19.0	14.1	11.9	13.4	10.6	10.7	10.3	8.0	6.5
median	6.9	5.0 ^a^	4.2	5.1 ^a^	2.7 ^b,c,d,e^	3.2 ^b,f^	2.4 ^c,f,g^	2.1 ^d,g^	2.6 ^e^
SD	36.9	25.9	23.5	24.1	22.8	21.7	22.6	20.7	15.4
minimum	0.2	0.2	0.2	0.2	0.2	0.2	0.2	0.2	0.2
maximum	357.6	422.8	313.0	237.6	218.9	315.8	247.4	161.5	315.9
IQR	14.2	12.3	9.7	11.2	7.1	8.0	6.8	5.8	4.6
90th%	48.3	36.2	29.1	36.2	28.1	27.3	28.1	16.4	13.0
95th%	81.2	59.6	49.1	59.6	52.4	47.9	54.6	29.5	23.5
abnormal	57.9%	49.8%	45.2%	50.4%	34.5%	38.9%	34.3%	31.5%	29.2%
normal	42.1%	50.2%	54.8%	49.6%	65.5%	61.1%	65.7%	68.5%	70.8%
<2 × ULN	18.2%	17.1%	17.5%	19.2%	12.8%	15.3%	13.8%	15.0%	15.5%
2-<3 × ULN	10.9%	8.9%	7.7%	9.0%	5.2%	6.2%	4.2%	6.0%	5.2%
3-<5 × ULN	11.5%	8.8%	8.2%	8.0%	5.3%	6.4%	5.9%	5.0%	3.9%
5-<10 × ULN	7.7%	8.6%	7.0%	8.0%	5.6%	6.2%	5.2%	3.0%	2.8%
10-<20 × ULN	6.1%	4.6%	3.3%	4.8%	4.2%	3.4%	4.1%	0.5%	1.3%
≥20 × ULN	3.6%	1.9%	1.6%	1.3%	1.3%	1.4%	1.1%	2.0%	0.5%

Note: Kruskal–Wallis H (df) = 1955.3 (8), *p* < 10^−6^. Pairwise comparisons (with Holm-adjusted *p*): gout versus any other (*p* < 10^−6^); RA versus PsA (*p* = 0.91) and RA versus the rest (*p* < 10^−14^); AS versus the rest (*p* < 10^−17^); PsA versus the rest (*p* < 10^−10^); SLE versus MCTD/SjD (*p* = 0.64), versus SS (*p* = 0.98), versus DM/PM (*p* = 0.69), and versus OA (*p* = 0.53); MCTD/SjD versus SS (*p* = 0.35), and versus the rest (*p* < 10^−9^); SS versus DM/PM (*p* = 0.98) and versus OA (*p* < 10^−10^). Non-significant associations are marked with corresponding letters (a–g). Abbreviations: AS—ankylosing spondylitis; CRP—C-reactive protein; IQR—interquartile range; MCTD—mixed connective tissue disease; OA—osteoarthritis; PsA—psoriatic arthritis; RA—rheumatoid arthritis; SD—standard deviation; SjD—Sjogren’s disease; SLE—systemic lupus erythematosus; SS—systemic sclerosis; ULN—upper limit of normal.

**Table 3:** Non-unique ESR distribution among diagnoses (*n* = 44,627 measurements).

	Gout	RA	AS	PsA	SLE	MCTD/SjD	SS	DM/PM	OA
*n*	811	15,579	6350	906	640	2886	521	193	17,844
mean	31.4	31.3	20.0	26.1	29.6	27.5	25.2	22.9	19.8
median	26.0 ^a^	25.0 ^a,b^	14.0	20.0 ^c,d^	25.0 ^b^	21.0 ^c,e,f^	20.0 ^d,e,g^	18.0 ^f,g^	16.0
SD	23.4	23.1	19.4	21.6	21.0	21.9	19.5	17.5	16.1
minimum	2.0	1.0	1.0	1.0	1.0	1.0	1.0	3.0	1.0
maximum	125.0	130.0	119.0	110.0	110.0	125.0	105.0	94.0	150.0
IQR	28.0	28.0	20.0	26.0	26.0	27.0	24.0	21.0	17.0
90th%	70.0	68.0	47.0	58.5	59.1	60.0	51.0	44.0	40.0
95th%	80.0	80.0	66.0	74.0	72.0	74.0	70.0	54.0	52.0
abnormal	61.4%	54.9%	37.1%	47.0%	55.3%	48.3%	45.7%	43.0%	32.2%
normal	38.6%	45.1%	62.9%	53.0%	44.7%	51.7%	54.3%	57.0%	67.8%
<2 × ULN	30.7%	29.3%	20.3%	25.7%	30.3%	27.0%	25.9%	28.0%	22.6%
2-<3 × ULN	13.8%	13.4%	8.5%	10.5%	15.8%	11.4%	12.5%	9.8%	6.2%
3-<5 × ULN	13.2%	11.0%	6.9%	9.7%	8.3%	8.2%	6.9%	4.1%	3.0%
5-<10 × ULN	3.7%	1.2%	1.5%	1.1%	0.9%	1.6%	0.4%	1.0%	0.4%

Note: Kruskal–Wallis H (df) = 3291.4 (8), *p* < 10^−6^. Pairwise comparisons (with Holm-adjusted *p*): gout versus RA (*p* = 0.96) and versus the rest (*p* < 10^−4^); RA versus SLE (*p* = 0.99) and versus the rest (*p* < 10^−6^); AS versus the rest (*p* < 10^−19^); PsA versus MCTD/SjD (*p* = 0.17), versus SS (*p* = 0.99), and versus the rest (*p* < 10^−5^); SLE versus the rest (*p* < 10^−3^); MCTD/SjD versus SS (*p* = 0.64), versus DM/PM (*p* = 0.17), and versus OA (*p* < 10^−9^); SS versus DM/PM (*p* = 0.99) and versus OA (*p* < 10^−9^). Non-significant associations are marked with corresponding letters (a–g). Abbreviations: AS—ankylosing spondylitis; ESR—erythrocyte sedimentation rate; IQR—interquartile range; MCTD—mixed connective tissue disease; OA—osteoarthritis; PsA—psoriatic arthritis; RA—rheumatoid arthritis; SD—standard deviation; SjD—Sjogren’s disease; SLE—systemic lupus erythematosus; SS—systemic sclerosis; ULN—upper limit of normal.

**Table 4:** Sex- and diagnosis-stratified correlations between CRP and ESR in same-day paired measurements.

	(a) Measurement Level		(b) Patient Level	
	*n* of CRP-ESR Pairs	ρ (CRP vs. ESR)	*n* of Patients with ≥1 CRP-ESR	ρ (Median CRP vs. Median ESR per Patient)
men	12,765	0.67	4515	0.64
women	31,624	0.58	12,309	0.53
gout	786	0.64	406	0.62
RA	15,495	0.63	3051	0.58
AS	6330	0.65	1106	0.65
PsA	1115	0.62	249	0.60
SLE	577	0.48	167	0.54
MCTD/SjD	2248	0.58	714	0.59
SS	342	0.44	80	0.46 *
DM/PM	92	0.70	26	0.65 ^#^
OA	17,133	0.48	10,935	0.47

Notes: Spearman rank correlations * *p* < 0.001; ^#^ *p* <0.01; for the rest, *p* < 10^−4^. Abbreviations: AS—ankylosing spondylitis; ESR—erythrocyte sedimentation rate; MCTD—mixed connective tissue disease; OA—osteoarthritis; PsA—psoriatic arthritis; RA—rheumatoid arthritis; SjD—Sjogren’s disease; SLE—systemic lupus erythematosus; SS—systemic sclerosis.

## Data Availability

The raw data supporting the conclusions of this article will be made available by the authors on reasonable request.

## Supplementary Materials

The following supporting information can be downloaded at https://www.mdpi.com/article/10.3390/clinpract16040072/s1. Table S1. Paired CRP-ESR results: baseline vs. overlap-excluded; Table S2. Patient-level CRP/ESR phenotypes by diagnosis (ever abnormal): baseline vs. overlap-excluded; Table S3. Paired CRP-ESR results: unweighted versus cluster bootstrap versus patient-weighted.

## Abbreviations

The following abbreviations are used in this manuscript:

AS	Ankylosing spondylitis
CRP	C-reactive protein
DM/PM	Dermatomyositis/polymyositis
ESR	Erythrocyte sedimentation rate
IQR	Interquartile range
KWH	Kruskal–Wallis H (test)
MCTD/SjD	Mixed connective tissue disease/Sjögren’s disease
MRI	Magnetic resonance imaging
MWU	Mann–Whitney U (test)
OA	Osteoarthritis
PsA	Psoriatic arthritis
RA	Rheumatoid arthritis
SD	standard deviation
SLE	Systemic lupus erythematosus
SS	Systemic sclerosis
ULN	Upper limit of normal

## References

[1] Spellberg B., Nielsen T.B., Phillips M.C., Ghanem B., Boyles T., Jegorović B., Footer B., Mah J.K., Lieu A., Scott J. (2025). Revisiting diagnostics: Erythrocyte sedimentation rate and C-reactive protein: It is time to stop the zombie tests. Clin. Microbiol. Infect..

[2] Desai R., Zhang R., Shah N., Bacalao M., Galous H., Karp D.R., Bajaj P. (2025). Reduction in the Concomitant Ordering of Erythrocyte Sedimentation Rate and C-Reactive Protein Within a Large Academic Medical Center. J. Gen. Intern. Med..

[3] Jackson K., Veillette J.J., Olson J., Seibert A.M., Webb B.J. (2025). Evaluation of serial erythrocyte sedimentation rate and C-reactive protein monitoring in infectious disease outpatient parenteral antimicrobial therapy patients. Antimicrob. Steward. Healthc. Epidemiol..

[4] Cho H.J., Talledo J., Alaiev D., Israilov S., Chandra K., Tsega S., Garcia M., Shin D.W., Zaurova M., Manchego P.A. (2023). Choosing Wisely and reducing the simultaneous ordering of erythrocyte sedimentation rate and C-reactive protein testing in a large safety net system. Am. J. Clin. Pathol..

[5] Parsaei A., Moradi S., Masoumi M., Davatchi F., Najafi A., Kooshki A.M., Hajighadery A., Akhlaghi M., Faezi T., Kavosi H. (2022). Predictive value of erythrocyte sedimentation rate and C-reactive protein in Behcet’s disease activity and manifestations: A cross-sectional study. BMC Rheumatol..

[6] Manzo C., Milchert M., Venditti C., Castagna A., Nune A., Natale M., Brzosko M. (2022). Fever Correlation with Erythrocyte Sedimentation Rate (ESR) and C-Reactive Protein (CRP) Concentrations in Patients with Isolated Polymyalgia Rheumatica (PMR): A Retrospective Comparison Study between Hospital and Out-of-Hospital Local Registries. Life.

[7] Park P.G., Song J.J., Park Y.B., Lee S.W. (2022). Clinical application of low erythrocyte sedimentation rate/high C-reactive protein to antineutrophil cytoplasmic antibody-associated vasculitis. J. Clin. Lab. Anal..

[8] Kuriya B., Schieir O., Lin D., Xiong J., Pope J., Boire G., Haraoui B., Thorne J.C., Tin D., Hitchon C. (2017). Thresholds for the 28-joint disease activity score (DAS28) using C-reactive protein are lower compared to DAS28 using erythrocyte sedimentation rate in early rheumatoid arthritis. Clin. Exp. Rheumatol..

[9] Son K.M., Kim S.Y., Lee S.H., Yang C.M., Seo Y.I., Kim H.A. (2016). Comparison of the disease activity score using the erythrocyte sedimentation rate and C-reactive protein levels in Koreans with rheumatoid arthritis. Int. J. Rheum. Dis..

[10] Tamhane A., Redden D.T., McGwin G., Brown E.E., Westfall A.O., Reynolds R.J., Hughes L.B., Conn D.L., Callahan L.F., Jonas B.L. (2013). Comparison of the disease activity score using erythrocyte sedimentation rate and C-reactive protein in African Americans with rheumatoid arthritis. J. Rheumatol..

[11] Song X., Wang Y.-H., Li M.-T., Duan X.-W., Li H.-B., Zeng X.-F., Guo L.-S. (2021). Chinese registry of rheumatoid arthritis: IV. Correlation and consistency of rheumatoid arthritis disease activity indices in China. Chin. Med. J..

[12] Firooz N., Albert D.A., Wallace D.J., Ishimori M., Berel D., Weisman M.H. (2011). High-sensitivity C-reactive protein and erythrocyte sedimentation rate in systemic lupus erythematosus. Lupus.

[13] Dima A., Opris D., Jurcut C., Baicus C. (2016). Is there still a place for erythrocyte sedimentation rate and C-reactive protein in systemic lupus erythematosus?. Lupus.

[14] Amezcua-Guerra L.M., Springall R., Arrieta-Alvarado A.A., Rodriguez V., Rivera-Martinez E., Castillo-Martinez D., Bojalil R. (2011). C-reactive protein and complement components but not other acute-phase reactants discriminate between clinical subsets and organ damage in systemic lupus erythematosus. Clin. Lab..

[15] Littlejohn E., Marder W., Lewis E., Francis S., Jackish J., McCune W.J., Somers E.C. (2018). The ratio of erythrocyte sedimentation rate to C-reactive protein is useful in distinguishing infection from flare in systemic lupus erythematosus patients presenting with fever. Lupus.

[16] Tsang H.H.L., Chung H.Y. (2017). The Discriminative Values of the Bath Ankylosing Spondylitis Disease Activity Index, Ankylosing Spondylitis Disease Activity Score, C-Reactive Protein, and Erythrocyte Sedimentation Rate in Spondyloarthritis-Related Axial Arthritis. J. Clin. Rheumatol..

[17] Queiro R., Alonso S., Burger S., Pardo E., Brana I., Loredo M., Alperi M. (2025). Tailoring Inflammatory Biomarker Assessment in Axial Spondyloarthritis: A Comparative Study of Erythrocyte Sedimentation Rate and C-Reactive Protein Across Disease Profiles. J. Pers. Med..

[18] Georgiadis S., Ornbjerg L.M., Michelsen B., Kvien T.K., Di Giuseppe D., Wallman J.K., Závada J., Provan S.A., Kristianslund E.K., Rodrigues A.M. (2024). Cut-Offs for Disease Activity States in Axial Spondyloarthritis With Ankylosing Spondylitis Disease Activity Score (ASDAS) Based on C-Reactive Protein and ASDAS Based on Erythrocyte Sedimentation Rate: Are They Interchangeable?. J. Rheumatol..

[19] Kara M., Alp G., Koc A.M. (2023). Diagnostic difficulties in polymyalgia rheumatica cases with normal erythrocyte sedimentation rate and C-reactive protein values. Medicine.

[20] Manzo C., Milchert M., Natale M., Brzosko M. (2019). Polymyalgia rheumatica with normal values of both erythrocyte sedimentation rate and C-reactive protein concentration at the time of diagnosis. Rheumatology.

[21] Alende-Castro V., Alonso-Sampedro M., Fernandez-Merino C., Sanchez-Castro J., Sopena B., Gude F., Gonzalez-Quintela A. (2021). C-Reactive Protein versus Erythrocyte Sedimentation Rate: Implications Among Patients with No Known Inflammatory Conditions. J. Am. Board. Fam. Med..

[22] Singh G. (2022). Re: C-Reactive Protein versus Erythrocyte Sedimentation Rate: Implications Among Patients with No Known Inflammatory Conditions. J. Am. Board Fam. Med..

[23] Costenbader K.H., Chibnik L.B., Schur P.H. (2007). Discordance between erythrocyte sedimentation rate and C-reactive protein measurements: Clinical significance. Clin. Exp. Rheumatol..

[24] Feldman M., Aziz B., Kang G.N., Opondo M.A., Belz R.K., Sellers C. (2013). C-reactive protein and erythrocyte sedimentation rate discordance: Frequency and causes in adults. Transl. Res..

[25] Torne Cachot J., Garcia Pont J., Camell Ilari H. (2022). Discrepancies between erythrocyte sedimentation rate and C-reactive protein in non-infectious inflammatory diseases. Med. Clin..

[26] Watson J., Jones H.E., Banks J., Whiting P., Salisbury C., Hamilton W. (2019). Use of multiple inflammatory marker tests in primary care: Using Clinical Practice Research Datalink to evaluate accuracy. Br. J. Gen. Pract..

