# Supplementary material for: Real-World Distributions and Concordance of C-Reactive Protein and Erythrocyte Sedimentation Rate Across Rheumatic Diseases
Source: Clin Pract. 2026 Apr 13;16(4):72. doi: 10.3390/clinpract16040072 (PMC13114268; doi:10.3390/clinpract16040072)
Supplement: Supplementary file 1 [file clinpract-16-00072-s001.zip › clinpract-4203972-supplementary.pdf]

Supplementary Table S1. Paired CRP-ESR results: baseline vs overlap-excluded

	Baseline	*Overlap-excluded*
Same-day CRP-ESR pairs	44427	41910
Unique patients with ≥1 pair	16824	16322
Spearman ρ (CRP vs ESR)	0.58	0.58
Spearman ρ (CRP category vs ESR category)	0.547	0.546
Both normal (CRP-/ESR-), %	45.4	46
CRP-/ESR+ only, %	15	14.7
CRP+/ESR- only, %	12.3	12.4
Both abnormal (CRP+/ESR+), %	27.3	26.9

Notes: Overlap-coded patients were defined as those with any laboratory record in the CRP or ESR indicator datasets where the sum of diagnosis indicators exceeded 1. Overlap-excluded analyses remove these patients from the paired CRP-ESR dataset. Spearman r correlations are shown; p values were $<10^{-4}$ for all correlations.

Abbreviations: CRP, C-reactive protein; ESR, erythrocyte sedimentation rate.

Supplementary Table S2. Patient-level CRP/ESR phenotypes by diagnosis (ever abnormal): baseline vs overlap-excluded

Diagnosis	Baseline n	Baseline CRP^+/ ESR^+	Baseline CRP^+/ ESR^-	Baseline CRP^-/ ESR^+	Baseline CRP^-/ ESR^-	Overlap -excluded n	Overlap -excluded CRP^+/ESR^+	Overlap-excluded CRP^+/ESR^-	Overlap-excluded CRP^-/ESR^+	Overlap-excluded CRP^-/ESR^-
AS	1009	56	14.6	6.6	22.8	990	55.9	14.7	6.7	22.7
DM/PM	28	28.6	3.6	25	42.9	24	25	4.2	25	45.8
Gout	413	52.5	12.1	13.3	22	365	50.1	12.6	13.7	23.6
MCTD/SjD	706	38.7	8.8	16.4	36.1	642	37.7	8.9	15.9	37.5
OA	11163	19	12.2	14.8	54	11063	18.9	12.2	14.8	54
PsA	228	53.1	15.4	10.1	21.5	215	53.5	16.3	10.2	20
RA	3010	65.1	9.2	11.7	14	2846	64.7	9.5	11.6	14.2
SLE	176	34.7	6.8	25.6	33	109	34.9	5.5	27.5	32.1
SS	91	35.2	11	16.5	37.4	68	33.8	11.8	14.7	39.7

Notes: Overlap-coded patients were defined as those with any laboratory record in the CRP or ESR indicator datasets where the sum of diagnosis indicators exceeded 1. Overlap-excluded analyses remove these patients from the paired CRP-ESR dataset. Patient-level phenotypes used an 'ever abnormal' rule across all paired measurements per patient; diagnosis was assigned from the first paired record (chronologically earliest) to create mutually exclusive patient strata.

Abbreviations: AS, ankylosing spondylitis; CRP, C-reactive protein; DM/PM, dermatomyositis/polymyositis; ESR, erythrocyte sedimentation rate; MCTD/SjD, mixed connective tissue disease/Sjögren's disease; OA, osteoarthritis; PsA, psoriatic arthritis; RA, rheumatoid arthritis; SLE, systemic lupus erythematosus; SS, systemic sclerosis; ULN, upper limit of normal.

Supplementary Table S3. Paired CRP-ESR results: unweighted versus cluster bootstrap versus patient-weighted

	Unweighted (point estimate)	*Cluster Bootstrap 95% CI*	*Patient-weighted estimate*
Same-day CRP–ESR pairs	44427	-	-
Unique patients with ≥1 pair	16824	-	-
Spearman r (CRP vs ESR)	0.580	0.566-0.594	0.549
Spearman r (CRP category vs ESR category)	0.547	0.534-0.560	0.509
CRP-/ESR-, % of paired measurements	45.4	44.4-46.4	50.9
CRP−/ESR+, % of paired measurements	15.0	14.3-15.5	15.1
CRP+/ESR−, % of paired measurements	12.3	11.8-12.8	12.1
CRP+/ESR+, % of paired measurements	27.3	26.6-28.0	21.8

Notes: Cluster bootstrap resampled patients with replacement (B=300), retaining all paired measurements per selected patient; 95% CI were computed using the 2.5th and 97.5th percentiles of bootstrap replicates. Patient-weighted estimates assign each patient equal total weight (weight=1) distributed across that patient's paired measurements (weight per pair = 1 / number of pairs for that patient). Spearman r values are shown as point estimates (unweighted), cluster bootstrap 95% CIs, and patient-weighted estimates.
Abbreviations: CRP, C-reactive protein; CI, confidence interval; ESR, erythrocyte sedimentation rate.
